# Einfluss der Corona-Pandemie auf Diagnostik und Therapie von Kopf-Hals-Tumoren

**DOI:** 10.1007/s00106-024-01520-0

**Published:** 2024-11-06

**Authors:** Benjamin Prokein, Michael Dau, Bernhard Frerich

**Affiliations:** Klinik und Poliklinik für Mund‑, Kiefer- und Plastische Gesichtschirurgie, Universitätsmedizin Rostock, Schillingallee 35, 18057 Rostock, Deutschland

**Keywords:** COVID-19, Oropharynxkarzinom, Tumorstadien, Deutschland, Mundhöhlenkarzinom, COVID-19, Oropharyngeal Carcinoma, Tumor stages, Germany, Oral cavity neoplasms

## Abstract

**Hintergrund:**

Nach Auftreten der ersten COVID-19-Fälle hatte sich das Virus innerhalb weniger Monate weltweit verbreitet. Hierbei zeigte sich ein Rückgang an ärztlichen Konsultationen. Die vorliegende Studie untersucht, ob dies Auswirkungen auf die Diagnose und Therapie von Kopf-Hals-Tumorerkrankungen hatte – bezogen auf den Zeitraum 2018 bis 2022.

**Material und Methode:**

Kopf-Hals-Tumordaten des Klinischen Krebsregisters (KKR) Mecklenburg-Vorpommern aus dem Zeitraum 2020–2022 wurden den beiden Vorjahren (2018, 2019) gegenübergestellt. Erfasst wurden demografische Daten, Fallzahlen, Daten zu Diagnose, ICD und die TNM-Klassifikation. Die COVID-19-Fälle wurden vom Robert Koch-Institut (RKI) abgefragt. Die Daten wurden mittels Mann-Whitney-U-Test und Korrelation nach Pearson analysiert.

**Ergebnisse:**

Insgesamt konnten 2332 Patientenfälle in die Studie eingeschlossen werden. Im Rahmen des Lockdowns zeigte sich weder ein signifikanter Rückgang der Tumormeldungen an das KKR noch eine signifikante Korrelation zwischen den COVID-19-Fallzahlen zu den Tumordiagnosen. Signifikante Unterschiede zeigten sich innerhalb der T‑Kategorie im Jahr 2022 zu den Prä-COVID-19-Jahren 2018 und 2019. Außerdem wurde eine Verschiebung der relativen Häufigkeiten der einzelnen ICD-10-Codes beobachtet.

**Schlussfolgerung:**

Im Rahmen der COVID-19-Pandemie zeigten sich keine signifikanten Unterschiede der Tumormeldungen beim Vergleich der Jahre 2018 bis 2022. Entgegen den Erwartungen eines Rückgangs der Fallzahlen in der Pandemie durch den Lockdown mit Erhöhung der Tumorstadien konnte sogar eine Reduktion der T‑Kategorie im Jahr 2022 gefunden werden sowie eine Verschiebung der relativen Häufigkeiten einzelner ICD-10-Codes über den Zeitraum der Pandemie.

## Abstract (en)

**Background:**

After the first appearance of COVID-19 cases, the virus spread worldwide within a few months. This led to a decrease in medical consultations. The present study investigates whether this effect had an impact on the diagnosis and treatment of head and neck cancer during the period from 2018 to 2022.

**Materials and methods:**

Data from the clinical cancer registration center (KKR) of Mecklenburg-Vorpommern from the 2020–2022 period were compared to those from the two previous years (2018, 2019). Demographic data, case numbers, diagnosis data, International Classification of Diseases (ICD) codes, and TNM classifications were recorded. COVID-19 case numbers were obtained from the Robert Koch Institute (RKI). Data were analyzed using the Mann–Whitney U test and Pearson’s correlation.

**Results:**

A total of 2332 patient cases could be included in this study. During the lockdown there was neither a significant reduction in tumor diagnoses registered at the KKR nor a correlation between COVID-19 case numbers and tumor diagnoses. Significant differences were found in terms of T category in the year 2022 compared to the pre-COVID years 2018 and 2019. Furthermore, there was a shift in the relative frequencies of some ICD-10 codes.

**Conclusion:**

During the COVID-19 pandemic, no significant differences in tumor diagnoses were observed upon comparing the years 2018 to 2022. Contrary to the expectation of a decrease in case numbers during the pandemic due to the lockdown with an increase in tumor stages, a reduction of the T category could be found in 2022, and a shift in the relative frequencies of some ICD-10 codes in the pandemic period was observed.

## Pandemiebedingte Einschränkungsmaßnahmen

Das „severe acute respiratory syndrome coronavirus 2“ (SARS-CoV-2) hatte sich innerhalb kurzer Zeit weltweit verbreitet. Viele Länder reagierten hierbei mit Einschränkungsmaßnahmen, um den hohen Inzidenzzahlen entgegenzuwirken. Studien aus der Inneren Medizin sowie der Notaufnahme ergaben rückläufige Fallzahlen an internistischen und chirurgischen Krankheitsbildern. Außerdem zeigten sich Auswirkungen auf die Diagnostik und Therapie von onkologischen Patienten. In dieser Studie sollen Auswirkungen der Coronapandemie auf Kopf-Hals-Tumorpatienten untersucht werden.

### Rückgang der Fallzahlen

Zu Beginn der Pandemie zeigte sich ein Rückgang an stationären Aufnahmen von Patienten mit Myokardinfarkt und durchgeführter perkutaner Intervention [5]. Weitere Studien zeigten einen Rückgang stationärer Aufnahmen von Patienten mit akutem Koronarsyndrom [4, 13]. Zudem wurde ein Rückgang an Patientenvorstellungen in Notaufnahmen beschrieben [14]. Als Ursachen werden unter anderem eine reduzierte Inanspruchnahme des Rettungsdienstes aus Angst vor COVID-19-Infektionen diskutiert [5, 7, 14].

### Auswirkungen auf Kopf-Hals-Tumorpatienten

In einer multizentrischen deutschen Studie zeigte sich die Inzidenz von Mundhöhlenkarzinomen nicht erhöht. Es wurde eine verringerte Zeit bis zur Intervention im Lockdown beschrieben [7]. Eine weitere Studie konnte keine signifikante Veränderung von T‑Kategorie, N‑Kategorie oder UICC-Stadium feststellen [1]. Andere Autoren konnten eine signifikant höhere Fernmetastasierungsrate sowie einen Anstieg an freien Lappentransplantaten im Lockdown nachweisen [10]. In Italien zeigten sich in der frühen Phase der COVID-19-Pandemie mehr fortgeschrittene Tumorstadien, wobei sich keine signifikante Therapieverzögerung nach Diagnostik feststellen ließ [11]. In den USA zeigte sich eine erhöhte primäre Tumorgröße mit weiter fortgeschrittener T‑Kategorie [9]. Diese Ergebnisse konnten auch in Österreich nachgewiesen werden [15]. In den Niederlanden zeigten sich im Jahr 2020 weniger Tumordiagnosen im Vergleich zu den Vorjahren. Dies hatte keinen Einfluss auf die Tumorstadien [17]. Weitere deutsche Studien wiesen heterogene Ergebnisse auf. In Bayern wurden in der frühen Phase der Pandemie weniger Tumordiagnosen festgestellt, welche allerdings ein weiter fortgeschrittenes Stadium zeigten. Zudem zeigte sich eine Verschiebung zu nichtchirurgischen Therapien. Im zweiten Jahr der Pandemie zeigten sich dann vermehrte Neudiagnosen [8]. In einer Studie aus Regensburg wurde kein Rückgang an Diagnosen oder Verschiebungen zu weiter fortgeschrittenen Tumorstadien gefunden [6].

In Heidelberg zeigte sich eine Verlängerung bis zum Therapiebeginn im Jahr 2020 mit signifikant höherer T‑Kategorie, während sich kein Einfluss auf die N‑Kategorie zeigte [12].

Carré et al. erfassten die Daten aus Brandenburg und Berlin vom Zeitraum 2018–2020. Hierbei verkürzte sich während der Pandemie der Zeitraum von Diagnose zu Therapiebeginn. Es wurde keine Zunahme der Tumorgröße oder des Tumorstadiums bei Erstvorstellung beobachtet [3].

Insgesamt zeigen sich national wie international heterogene Ergebnisse, welche auch in Abhängigkeit von der Lokalisation des Studienortes mit den doch stark unterschiedlichen politischen Vorgehensweisen und Einschränkungsmaßnahmen des jeweiligen Landes stehen. Mit dieser Studie soll untersucht werden, ob sich die Pandemie sowie der Lockdown auf die Diagnostik und damit verbunden auf die Therapie von Kopf-Hals-Tumorpatienten in Mecklenburg-Vorpommern ausgewirkt hat, anhand des großen Patientenkollektivs des Klinischen Krebsregisters Mecklenburg-Vorpommern (KKR M‑V) über die Zeitperiode von 2018 bis 2022.

## Studiendesign und Untersuchungsmethoden

Alle Patienten mit einem Kopf-Hals-Karzinom, welches im Zeitraum 2018 bis 2022 im Klinischen Krebsregister Mecklenburg-Vorpommern registriert wurde, wurden in die Studie inkludiert. Die Abfrage der Daten vom Krebsregister erfolgte im Dezember 2023. Im März 2024 wurden diese übermittelt. Es gab keine Ausschlusskriterien. Die ICD-10-Codes C00–C14 wurden eingeschlossen [2]. Es wurden TNM-Klassifikation (sowohl cTNM sowie pTNM), Diagnose, Lokalisation, ICD-10-Code, Geschlecht und Alter erfasst. Die Daten waren hierbei pseudoanonymisiert (Identifikationsnummer). Darüber hinaus wurden die COVID-19-Fälle in Deutschland im Jahr 2020 durch das Robert Koch-Institut (RKI) in Kalenderwochen erfasst [16]. Es erfolgte die Ermittlung der Fallzahlen für die jeweiligen Kalenderjahre in Kalenderwochen. Zunächst wurde eine Auswertung der demografischen sowie tumorspezifischen Daten durchgeführt. Die Datensätze von 2020–2022 wurden denen der beiden Vorjahre (2018, 2019) gegenübergestellt. Hierbei wurden die TNM-Kategorien des KKR von 2018–2022 zunächst auf Normalverteilung mittels Shapiro-Wilk-Test überprüft und aufgrund von nicht vorhandener Normalverteilung mittels Mann-Whitney-U-Test ausgewertet. Zusatzbeschreibungen in Form von Buchstaben nach der Zahl (z. B. T4a) wurden hierbei entfernt, um rein numerische Werte zu ermitteln. Außerdem erfolgte eine Korrelation nach Pearson der Fallzahlen von 2020 mit den gemeldeten COVID-19-Fallzahlen des RKI. Die Fallzahlen je Kalenderwoche der unterschiedlichen Kalenderjahre wurden auf Normalverteilung mittels Shapiro-Wilk-Test überprüft und aufgrund fehlender Normalverteilung mittels Mann-Whitney-U-Test verglichen. *p*-Werte von *p* ≤ 0,05 wurden als signifikant angesehen. Die statistische Auswertung wurde mittels IBM (Böblingen, Deutschland) SPSS Statistics (Version: 28.0.1.0) durchgeführt.

## Ergebnisse

Insgesamt konnten 2332 Fälle in die Studie eingeschlossen werden. Betrachtet man die Fallzahlen über die Jahre 2018–2022, so zeigt sich ein weitestgehend konstantes Niveau dieser um 20 % je Kalenderjahr. Das Alter lag hierbei zwischen 21 und 100 Jahren mit einem Mittelwert von 64,4 Jahren. Der überwiegende Teil im untersuchten Patientengut war männlich (75,3 %), was sich relativ konstant über die Kalenderjahre 2018–2022 zeigte. Die am häufigsten registrierten ICD-10-Codes waren C09.- (Bösartige Neubildung der Tonsille; 414 Meldungen) mit einem Anteil von 17,8 % sowie C02.- (Bösartige Neubildung sonstiger und nicht näher bezeichneter Teile der Zunge; 308 Meldungen) mit 13,2 % sowie C04.- (Bösartige Neubildung des Mundbodens; 290 Meldungen) mit 12,4 %. Betrachtet man die Fallzahlen der einzelnen ICD-10-Codes im zeitlichen Verlauf, zeigt sich über die Jahre 2018–2022 eine Verdopplung der C00.-Fälle (Bösartige Neubildung der Lippe) von 13 auf 27 Meldungen mit einem Anstieg des relativen Anteils von 2,7 % auf 6,1 % sowie mit einem Anstieg der C07.-Fälle (Bösartige Neubildung der Parotis) von 2,5 % (12 Fälle) 2018 auf ein Maximum 2020 mit 4,8 % (23 Fälle) und zuletzt einem leichten Rückgang auf 3,9 % (17 Fälle). Ein Rückgang der Fallzahlen verzeichnete sich bei C10.- (Bösartige Neubildung des Oropharynx) von 12,8 % (61 Fälle) auf 6,9 % (32 Fälle) 2021 mit einem zuletzt leichten Anstieg 2022 auf 9,1 % (40 Fälle). Ebenso sank die Anzahl an C13.- (Bösartige Neubildung des Hypopharynx) im zeitlichen Verlauf von 9,4 % (45 Fälle) auf 6,6 % (29 Fälle) im Jahr 2022. Bei den anderen ICD-10-Codes zeigte sich ein weitestgehend konstantes bzw. leicht undulierendes Niveau. Details sind in Tab. 1 dargestellt.

**Tab. 1:** Demografische Daten

	Absolute Häufigkeit (*n* = 2332)					
	Gesamt	2018	2019	2020	2021	2022
Fallzahlen	2332 (100 %)	477 (20,5 %)	475 (20,4 %)	476 (20,4 %)	464 (19,9 %)	440 (18,9 %)
Alter (Jahre)						
Min.	21	23	26	21	36	31
Max.	100	92	94	95	96	100
Mittelwert	64,4	63,3	63,7	64,6	65,3	65,4
Geschlecht						
männlich	1755 (75,3 %)	361 (75,7 %)	368 (77,5 %)	359 (75,4 %)	337 (72,6 %)	330 (75,0 %)
weiblich	577 (24,7 %)	116 (24,3 %)	107 (22,5 %)	117 (24,6 %)	127 (27,4 %)	110 (25,0 %)
ICD-10-Code						
C00.-	106 (4,5 %)	13 (2,7 %)	21 (4,4 %)	17 (3,6 %)	26 (5,6 %)	27 (6,1 %)
C01.-	148 (6,3 %)	34 (7,1 %)	26 (5,5 %)	26 (5,5 %)	35 (7,5 %)	27 (6,1 %)
C02.-	308 (13,2 %)	65 (13,6 %)	57 (12,0 %)	64 (13,4 %)	66 (14,2 %)	56 (12,7)
C03.-	144 (6,2 %)	27 (5,7 %)	18 (3,8 %)	40 (8,4 %)	33 (7,1 %)	26 (5,9 %)
C04.-	290 (12,4 %)	60 (12,6 %)	66 (13,9 %)	55 (11,6 %)	59 (12,7 %)	50 (11,4 %)
C05.-	140 (6,0 %)	29 (6,1 %)	25 (5,3 %)	30 (6,3 %)	27 (5,8 %)	29 (6,6 %)
C06.-	84 (3,6 %)	16 (3,4 %)	16 (3,4 %)	15 (3,2 %)	22 (4,7 %)	15 (3,4 %)
C07.-	83 (3,6 %)	12 (2,5 %)	12 (2,5 %)	23 (4,8 %)	19 (4,1 %)	17 (3,9 %)
C08.-	28 (1,2 %)	6 (1,3 %)	3 (0,6 %)	9 (1,9 %)	4 (0,9 %)	6 (1,4 %)
C09.-	414 (17,8 %)	76 (15,9 %)	107 (22,5 %)	73 (15,3 %)	74 (15,9 %)	84 (19,1 %)
C10.-	236 (10,1 %)	61 (12,8 %)	58 (12,2 %)	45 (9,5 %)	32 (6,9 %)	40 (9,1 %)
C11.-	38 (1,6 %)	9 (1,9 %)	3 (0,6 %)	10 (2,1 %)	7 (1,5 %)	9 (2,0 %)
C12.-	93 (4,0 %)	23 (4,8 %)	16 (3,4 %)	21 (4,4 %)	18 (3,9 %)	15 (3,4 %)
C13.-	213 (9,1 %)	45 (9,4 %)	47 (9,9 %)	46 (9,7 %)	37 (8,0 %)	29 (6,6 %)
C14.-	7 (0,3 %)	1 (0,2 %)	0 (0,0 %)	2 (0,4 %)	3 (0,6 %)	1 (0,2 %)

*C00.-* Bösartige Neubildung der Lippe; *C01.-* Bösartige Neubildung des Zungengrundes; *C02.-* Bösartige Neubildung sonstiger und nicht näher bezeichneter Teile der Zunge; *C03.-* Bösartige Neubildung des Zahnfleisches; *C04.-* Bösartige Neubildung des Mundbodens; *C05.-* Bösartige Neubildung des Gaumens; *C06.-* Bösartige Neubildung sonstiger und nicht näher bezeichneter Teile des Mundes; *C07.-* Bösartige Neubildung der Parotis; *C08.-* Bösartige Neubildung sonstiger und nicht näher bezeichneter großer Speicheldrüsen; *C09.-* Bösartige Neubildung der Tonsille; *C10.-* Bösartige Neubildung des Oropharynx; *C11.-* Bösartige Neubildung des Nasopharynx; *C12.-* Bösartige Neubildung des Recessus piriformis; *C13.-* Bösartige Neubildung des Hypopharynx; *C14.-* Bösartige Neubildung sonstiger und ungenau bezeichneter Lokalisationen der Lippe, der Mundhöhle und des Pharynx

**Abb. 1:**
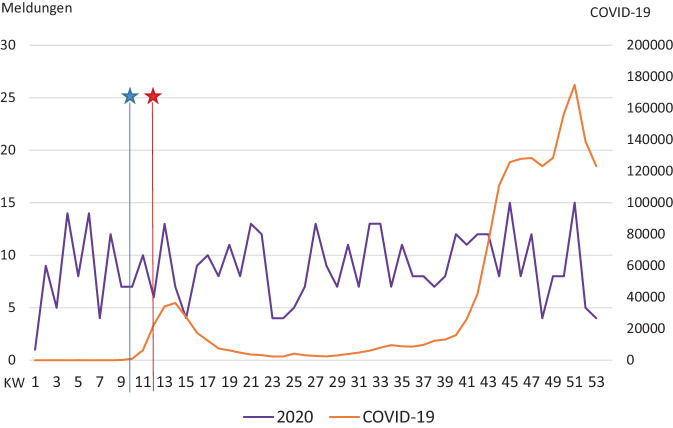
Fallzahlen. *KW* Kalenderwoche, *Blauer Stern *1. COVID-19-Fall in Mecklenburg-Vorpommern; *roter Stern *1. Lockdown. Die Abbildung zeigt die Anzahl an Meldungen von Tumordiagnosen an das KKR des Jahres 2020 sowie im Verhältnis hierzu die gemeldeten COVID-19-Fallzahlen. Im Rahmen des 1. Lockdowns zeigt sich ein Rückgang der Tumordiagnosen im Jahr 2020 bei korrespondierend initial steigenden und anschließend wieder rasch abfallenden COVID-19-Fallzahlen. Außerdem zeigt sich ein erneutes Tief im Bereich der 23.–25. Kalenderwoche, ohne dass es hierbei zu einem Anstieg der COVID-19-Fallzahlen kam. Der Anstieg der COVID-19-Fallzahlen gegen Ende des Jahres hatte keinen Einfluss auf die Tumordiagnosen

Das RKI meldete am 04.03.2020 (10. Kalenderwoche) den ersten COVID-19-Fall in Mecklenburg-Vorpommern. Zur Eindämmung der Infektion wurde am 17.03.2020 (12. Kalenderwoche) der erste Lockdown beschlossen. In Abb. 1 sind die Fallzahlen des KKR des Jahres 2020 sowie die deutschlandweiten COVID-19-Fallzahlen 2020 aufgeführt. Bei initial steigenden COVID-19-Meldungen zeigt sich ein leichter Einbruch der Fallmeldungen an das KKR um die 15. Kalenderwoche. Außerdem zeigt sich ein erneutes Tief im Bereich der 23.–25. Kalenderwoche. Der Bereich der ersten drei Monate nach dem Lockdown wurde in der Studie als „frühe Lockdown-Phase“ (Kalenderwochen 12–25, 16.03.2020–24.06.2020) definiert. Bei der Korrelation der COVID-19-Fälle mit den gemeldeten Fällen 2020 an das KKR mittels Pearson-Korrelation zeigte sich kein signifikanter Zusammenhang (*p* = 0,698).

Die Fallzahlen des KKR waren im Shapiro-Wilk-Test nicht normalverteilt (*p* = 0,039). Beim Vergleich der Fallzahlen der Kalenderwochen 12–25 (frühe Lockdown-Phase) aus der „Prä-COVID-19-Ära“ (2018, 2019) mit den Fallzahlen der „COVID-19-Ära“ (2020, 2021, 2022) zeigten sich im Mann-Whitney-U-Test keine signifikanten Unterschiede (Tab. 2).

**Tab. 2:** Fallzahlen früher Lockdown

	2018	2019	2020	2021	2022	2018/2019
2018	–	*p* = 0,743	*p* = 0,864	*p* = 0,656	*p* = 0,811	–
2019	*p* = 0,743	–	*p* = 0,739	*p* = 0,293	*p* = 0,638	–
2020	*p* = 0,864	*p* = 0,739	–	*p* = 0,812	*p* = 0,883	–
2021	*p* = 0,656	*p* = 0,293	*p* = 0,812	–	*p* = 0,845	–
2022	*p* = 0,811	*p* = 0,638	*p* = 0,883	*p* = 0,845	–	–
2020/2021/2022	–	–	–	–	–	*p* = 0,469

Überprüft wurden die Fallzahlen der Tumormeldungen an der KKR der Jahre 2018 bis 2022 im Zeitraum des frühen Lockdowns (KW 12–25) mittels Mann-Whitney-U-Test. Hierbei konnten keine signifikanten Unterschiede festgestellt werden

Außerdem wurden die TNM-Kategorien über die Kalenderjahre 2018–2022 untersucht. Zunächst wurde für jede Kategorie eine Überprüfung der Kalenderjahre auf Normalverteilung mittels Shapiro-Wilk-Test durchgeführt. Für jede Kategorie zeigten sich die Daten hierbei nicht normalverteilt (T-Kategorie *p* < 0,001; N‑Kategorie *p* < 0,001; M‑Kategorie *p* < 0,001).

Während sich bei den N‑ und M‑Kategorien im Mann-Whitney-U-Test keine Signifikanzen ergaben, zeigten sich signifikante Unterschiede in der T‑Kategorie zwischen dem Jahr 2022 jeweils zu den Prä-COVID-19-Jahren 2018 (U = 78503,000; Z = −2,040; *p* = 0,041) und 2019 (U = 80660,000; Z = −2,048; *p* = 0,041) sowie auch beim Vergleich der Prä-COVID-19-Ära zu der COVID-19-Ära (U = 535593,000; Z = −2,371; *p* = 0,018).

Die Werte sind in Tab. 3, 4, 5 und 6 dargestellt. Außerdem zeigte sich bei der T‑Kategorie in der Phase des frühen Lockdowns beim Vergleich der Jahre 2018 zu 2022 ebenfalls ein signifikanter Unterschied (*p* = 0,021). In Tab. 3 sind die Häufigkeiten der einzelnen TNM-Kategorien für die Jahre 2018–2022 dargestellt. Erkennbar ist die Erhöhung des relativen Anteils niedriger T‑Kategorien insbesondere von T1 im Jahr 2022 auf 35,4 %, während ein Rückgang bei T2 und T3 gefunden wurde im Vergleich zum Durchschnittswert der 5 Kalenderjahre.

**Tab. 3:** TNM-Kategorien

	Absolute Häufigkeit (*n* = 2219)					
	Gesamt	2018	2019	2020	2021	2022
Fallzahlen	2219 (100 %)	443 (20,0 %)	457 (20,6 %)	468 (21,1 %)	447 (20,1 %)	404 (18,2 %)
T1	664 (29,9 %)	123 (27,8 %)	113 (24,7 %)	144 (30,8 %)	141 (31,5 %)	143 (35,4 %)
T2	551 (24,8 %)	113 (25,5 %)	129 (28,2 %)	110 (23,5 %)	112 (25,1 %)	87 (21,5 %)
T3	386 (17,4 %)	72 (16,3 %)	94 (20,6 %)	78 (16,7 %)	84 (18,8 %)	58 (14,4 %)
T4	569 (25,6 %)	128 (28,9 %)	112 (24,5 %)	127 (27,1 %)	99 (22,1 %)	103 (25,5 %)
Fehlend	49 (2,2 %)	7 (1,6 %)	9 (2,0 %)	9 (1,9 %)	11 (2,5 %)	13 (3,2 %)
N0	1051 (47,4 %)	211 (47,6 %)	204 (44,6 %)	226 (48,3 %)	222 (49,7 %)	188 (46,5 %)
N1	322 (14,5 %)	62 (14,0 %)	79 (17,3 %)	65 (13,9 %)	62 (13,9 %)	54 (13,4 %)
N2	557 (25,1 %)	118 (26,6 %)	117 (25,6 %)	126 (26,9 %)	101 (22,6 %)	95 (23,5 %)
N3	215 (9,7 %)	41 (9,3 %)	42 (9,2 %)	38 (8,1 %)	49 (11,0 %)	45 (11,1 %)
N4	1 (0,0 %)	–	–	1 (0,2 %)	–	–
Fehlend	73 (3,3 %)	11 (2,4 %)	15 (3,3 %)	12 (2,6 %)	13 (2,9 %)	22 (5,4 %)
M0	2051 (92,4 %)	419 (94,6 %)	422 (92,3 %)	436 (93,2 %)	414 (92,6 %)	360 (89,1 %)
M1	77 (3,5 %)	13 (2,9 %)	15 (3,3 %)	14 (3,0 %)	16 (3,6 %)	19 (4,7 %)
Fehlend	91 (4,1 %)	11 (2,5 %)	20 (4,4 %)	18 (3,8 %)	17 (3,8 %)	25 (6,2 %)

Häufigkeiten der TNM-Kategorien der Jahre 2018 bis 2022 im Vergleich. Aufgrund unvollständiger Datensätze der TNM-Kategorien ist die Gesamtzahl der hierbei berücksichtigen Fälle auf 2219 reduziert. Zusatzbezeichnungen in Form von Buchstaben nach der Zahl wurden entfernt, um rein numerische Werte zu erhalten. Diese sind dann in der numerischen Kategorie zusammengefasst (z. B. T4a, T4b = T4). Unvollständige Angaben sowie die Bezeichnung „X“ nach der Kategorie sind unter „fehlend“ zusammengefasst. Einmalig wurde 2020 die Angabe N4 erfasst, was als Übertragungsfehler interpretiert wurde

**Tab. 4:** T‑Kategorie

	2018	2019	2020	2021	2022	2018/2019
2018	–	*p* = 0,930	*p* = 0,421	*p* = 0,066	***p*** **=** **0,041**	–
2019	*p* = 0,930	–	*p* = 0,457	*p* = 0,066	***p*** **=** **0,041**	–
2020	*p* = 0,421	*p* = 0,457	–	*p* = 0,301	*p* = 0,200	–
2021	*p* = 0,066	*p* = 0,066	*p* = 0,301	–	*p* = 0,740	–
2022	***p*** **=** **0,041**	***p*** **=** **0,041**	*p* = 0,200	*p* = 0,740	–	–
2020/2021/2022	–	–	–	–	–	***p*** **=** **0,018**

Die Auswertung erfolgte mittels Mann-Whitney-U-Test der Kategorie T der beobachteten Jahre 2018 bis 2022. Es zeigten sich hierbei signifikante Unterschiede beim Vergleich des Jahres 2022 zu den Prä-COVID-19-Jahren 2018 und 2019 (fett hervorgehoben)

**Tab. 5:** N‑Kategorie

	2018	2019	2020	2021	2022	2018/2019
2018	–	*p* = 0,710	*p* = 0,773	*p* = 0,633	*p* = 0,886	–
2019	*p* = 0,710	–	*p* = 0,502	*p* = 0,380	*p* = 0,823	–
2020	*p* = 0,773	*p* = 0,502	–	*p* = 0,839	*p* = 0,675	–
2021	*p* = 0,633	*p* = 0,380	*p* = 0,839	–	*p* = 0,554	–
2022	*p* = 0,886	*p* = 0,823	*p* = 0,675	*p* = 0,554	–	–
2020/2021/2022	–	–	–	–	–	*p* = 0,521

Die Auswertung erfolgte mittels Mann-Whitney-U-Test der Kategorie N der beobachteten Jahre 2018 bis 2022. Es zeigten sich hierbei keine signifikanten Unterschiede

**Tab. 6:** M‑Kategorie

	2018	2019	2020	2021	2022	2018/2019
2018	–	*p* = 0,848	*p* = 1,00	*p* = 0,577	*p* = 0,152	–
2019	*p* = 0,848	–	*p* = 0,851	*p* = 0,857	*p* = 0,294	–
2020	*p* = 1,00	*p* = 0,851	–	*p* = 0,711	*p* = 0,212	–
2021	*p* = 0,577	*p* = 0,857	*p* = 0,711	–	*p* = 0,391	–
2022	*p* = 0,152	*p* = 0,294	*p* = 0,212	*p* = 0,391	–	–
2020/2021/2022	–	–	–	–	–	*p* = 0,479

Die Auswertung erfolgte mittels Mann-Whitney-U-Test der Kategorie M der beobachteten Jahre 2018 bis 2022. Es zeigten sich hierbei keine signifikanten Unterschiede

## Diskussion

Durch die Daten des KKR konnte auf ein großes Patientenkollektiv von 2332 Patientenfällen zurückgegriffen werden.

Es zeigte sich hierbei die Beobachtung, dass es innerhalb der unterschiedlichen ICD-10-Codes zu einer Dynamik einzelner ICD-10-Kategorien kam. Interessant wäre, hier den Verlauf der kommenden Jahre weiter zu beobachten, um langfristige Trends zu erfassen oder aber dies als Phänomen der COVID-19-Pandemie zu interpretieren. Über die Gründe hierzu kann zum aktuellen Zeitpunkt nur spekuliert werden, da die auffälligen Entitäten (Lippe, Parotis, Oropharynx, Hypopharynx) keine offensichtlichen Gemeinsamkeiten aufweisen, welche durch die Pandemie erklärbar wären.

Entgegen der Ergebnisse anderer Studien [8, 17] zeigte sich in dem Patientenkollektiv dieser Studie kein signifikanter Rückgang der Patientenfälle im Rahmen der COVID-19-Pandemie. Dies deckt sich mit den Ergebnissen anderer Autoren [1, 6, 7]. Auffällig ist die Divergenz der unterschiedlichen Ergebnisse in Abhängigkeit von der geografischen Lage. Gegebenenfalls haben hier regionale Unterschiede beim Umgang mit den Einschränkungsmaßnahmen oder auch die Bevölkerungsdichte je Studienregion eine Rolle gespielt, welche das Verhalten der Menschen beeinflusst haben. Dies erklärt allerdings nicht die unterschiedlichen Ergebnisse innerhalb Bayerns [6, 8].

In Abb. 1 sind die Fallzahlen der Tumordiagnosen des KKR sowie von COVID-19 des RKI im Jahresverlauf dargestellt. Man erkennt nach Auftreten des 1. COVID-19-Falls in Mecklenburg-Vorpommern den raschen Anstieg der Fallzahlen, welche nach Ausrufen des ersten Lockdowns wieder rückläufig waren. Zeitgleich kam es zum Rückgang der Patientenfälle in den folgenden Wochen. Als es gegen Ende des Jahres 2020 zu einem erneuten starken Anstieg der COVID-19-Fallzahlen kam, hatte dies keine Auswirkungen mehr auf die Meldungen. In der Literatur werden insbesondere am Anfang der Pandemie zurückhaltende Konsultationen in Krankenhäusern beschrieben. Als mögliche Ursache werden individuelle Ängste vor Ansteckungen im Krankenhaus sowie an öffentlichen Plätzen sowie eine generelle Verunsicherung diskutiert [5, 7, 14].

Im Zeitraum des Lockdowns im Jahr 2020 zeigte sich keine signifikante Korrelation zwischen den COVID-19-Fallzahlen zu den Patientenfällen, im Gegensatz zu anderen Studien, die im Rahmen der chirurgischen Notaufnahme eine negative Korrelation zwischen COVID-19-Fallzahlen und Fallzahlen der Notaufnahme nachweisen konnten [14].

Im Rahmen der Untersuchung der TNM-Kategorien ergab sich in dieser Studie ein signifikanter Unterschied bei der T‑Kategorie zwischen dem Jahr 2022 zu den Prä-COVID-19-Jahren 2018 und 2019. Während man Unterschiede vor allem am Anfang der Pandemie erwartet, treten diese erst 2022 auf und zeigen einen Rückgang innerhalb der T‑Kategorie. Da keine weiteren Daten über den Pandemiezeitraum bis 2022 anderer Autoren vorliegen, kann keine Aussage darüber getroffen werden, ob dies ein lokales Phänomen innerhalb Mecklenburg-Vorpommerns darstellt oder ob sich dies auch in anderen Regionen so zeigt.

Heimes et al. konnten in ihrer Studie keinen signifikanten Unterschied bezüglich der Anzahl maligner Erkrankungen im Zeitraum der COVID-19-Pandemie finden. Dies deckt sich mit den Ergebnissen dieser Studie, wo sich eine homogene Anzahl der Tumordiagnosen verteilt über die Jahre 2018–2022 zeigte. Entgegen ersten Vermutungen ergab sich hier sogar eine verkürzte Zeit bis zur Intervention, sodass es während der Lockdown-Phase nicht zu einer befürchteten verzögerten Therapie kam. Dies kann als Erklärungsansatz auch für diese Studie angewandt werden, wo sich keine Erhöhung der TNM-Kategorien zeigte, wobei sogar 2022 ein signifikanter Rückgang der T‑Kategorie gefunden wurde [7].

Eine monozentrische Studie von Balk et al. mit 612 Patientenfällen beschreibt, wie auch die vorliegende Studie, dass es zu keinem signifikanten Rückgang an Tumordiagnosen im Rahmen der COVID-19-Pandemie kam. Während sich T‑ und N‑Kategorie ebenfalls nicht unterschieden, zeigten sich mehr Patienten mit Fernmetastasen (M+) im Prä-COVID-19-Zeitraum [1]. Entgegen den initialen Erwartungen von verspätetem Therapiebeginn sowie folglich dem Anstieg von Fernmetastasen konnten in der vorliegenden Studie keine signifikanten Unterschiede hinsichtlich der N‑ und M‑Kategorie gefunden werden.

In Bayern fanden sich in der frühen Pandemie signifikant mehr fortgeschrittene Tumorstadien im Vergleich zu den Vorjahren sowie ein Aufholeffekt an Fallzahlen zu Beginn des Jahres 2021 [8]. Dies konnte in dieser Studie nicht festgestellt werden, wo sich initial kein Unterschied innerhalb der TNM-Kategorien fand, und widerspricht auch den Daten anderer Autoren [1, 6]. So zeigt sich in einer weiteren Studie ebenfalls aus Bayern (Regensburg) weder ein Rückgang an Tumordiagnosen noch eine Verschiebung innerhalb der TNM-Kategorien [6].

Metzger et al. konnten in Heidelberg eine Behandlungsverzögerung 2020 mit erhöhter T‑Kategorie in 2020 ohne Änderung der N‑Kategorie feststellen [12]. Dies deckt sich nicht mit diesem Studienkollektiv, wo sich innerhalb der T‑Kategorie keine Erhöhung fand, wobei hinsichtlich der N‑Kategorie gleiche Ergebnisse gefunden wurden.

Ebenfalls zeigen Daten aus Brandenburg und Berlin, dass keine Zunahme der Tumorstadien gefunden wurde. Da sich diese Daten jedoch nur auf 2020 beziehen, ist ein eventueller folgender Anstieg 2021 oder 2022 nicht beurteilbar [3]. So konnte in dem Kollektiv der vorliegenden Studie erst im Jahr 2022 ein Unterschied innerhalb der T‑Kategorie festgestellt werden.

Vergleicht man die Ergebnisse der vorliegenden Studien, zeigen sich international, aber vor allem auch national Unterschiede hinsichtlich des Einflusses der COVID-19-Pandemie auf Kopf-Hals-Tumoren. Regionale Unterschiede beim Umgang mit den Einschränkungsmaßnahmen und regionalen COVID-19-Fallzahlen müssen hierbei als Erklärungsmodell diskutiert werden, wobei sich sogar innerhalb Bayers diskrepante Ergebnisse zeigen.

Im Rahmen dieser Studie wurden die COVID-19-Fallzahlen des RKI innerhalb Deutschlands herangezogen, da die totalen deutschlandweiten Fallzahlen die politische Stimmung innerhalb Deutschlands maßgebend prägten. Als Flächenland wurden in Mecklenburg-Vorpommern zum Teil unterschiedliche Anstiege und Dynamiken im Vergleich zu Ballungszentren wie z. B. Berlin beschrieben, was ggf. die Aussagekraft der Korrelation der Patientenfälle inhibiert. Außerdem wurden sowohl cTNM- wie auch pTNM-Daten eingeschlossen, um auch primär radio(chemo)therapeutisch behandelte Fälle zu inkludieren. Eine Aufschlüsselung der Daten mit Unterscheidung zwischen cTNM und pTNM würde die Aussagekraft dieser Studie erhöhen.

Der Bundesgesundheitsminister Karl Lauterbauch erklärte im April 2023 die COVID-19-Pandemie für beendet. Im Rahmen dieser Studie konnte mit dem Einschluss der Jahre 2018 bis 2022 fast der komplette Pandemie-Zeitrahmen erfasst werden. Die Daten für 2023 lagen zum Zeitpunkt der Abfrage an das KKR noch nicht vor. Eine zusätzliche Abbildung der Daten für 2023 wäre wünschenswert, um die Pandemie vollumfänglich abzubilden.

## Ausblick

Zusammenfassend lässt sich sagen, dass keine einheitliche Aussage für Deutschland getroffen werden kann. Mit dieser Studie konnte erstmals eine prolongierte Darstellung des Einflusses der COVID-19-Pandemie untersucht werden, welche fast den kompletten Zeitraum der definierten Pandemie erfasst und damit auch eventuelle „Aufholeffekte“ mit abbildet. Weitere Studien sind sinnvoll, um den Einfluss in weiteren Regionen Deutschlands sowie auch international für den Zeitraum der kompletten Pandemie darzustellen und den Ergebnissen aus Deutschland gegenüberzustellen. Entgegen ersten Vermutungen, dass die Pandemie sowie insbesondere der Lockdown zu einer Verschiebung zu höheren Tumorstadien führte, zeigte sich in dem Studienkollektiv kein negativer Einfluss auf die TNM-Kategorie von Kopf-Hals-Tumorpatienten, wobei sich 2022 sogar ein signifikanter Rückgang innerhalb der T‑Kategorie zeigte. Es konnte eine Verschiebung der Häufigkeiten einzelner ICD-10-Codes beobachtet werden über den Zeitraum der Pandemie. Innerhalb Deutschlands zeigten sich divergierende Ergebnisse des Einflusses der COVID-19-Pandemie auf Kopf-Hals-Tumorpatienten.

## Data Availability

Die Originalrohdaten wurden durch das KKR M‑V bereitgestellt und können bei Bedarf dort angefragt werden.
